# Oral cancer associated with chronic mechanical irritation of the oral mucosa

**DOI:** 10.4317/medoral.22017

**Published:** 2018-02-25

**Authors:** Eduardo Piemonte, Jerónimo Lazos, Paola Belardinelli, Dante Secchi, Mabel Brunotto, Hector Lanfranchi-Tizeira

**Affiliations:** 1 DDS, PhD. Associate Professor, Oral Medicine Department, Dentistry College, Universidad Nacional de Córdoba, Córdoba, Argentina; 2 DDS. Cert. Perio. Assistant Professor, Oral Medicine Department, Dentistry College, Universidad Nacional de Córdoba, Córdoba, Argentina; 3 DDS, PhD. Assistant Professor, Oral Medicine Department, Dentistry College, Universidad Nacional de Córdoba, Córdoba, Argentina; 4 PhD, MSc. Head Professor, Oral Biology Department, Dentistry College, Universidad Nacional de Córdoba, Córdoba, Argentina; 5 DDS, PhD. Head Professor, Oral Medicine Department, Dentistry College, Universidad de Buenos Aires, CABA, Argentina

## Abstract

**Background:**

Most of the studies dealing with Chronic Mechanical Irritation (CMI) and Oral Cancer (OC) only considered prosthetic and dental variables separately, and CMI functional factors are not registered. Thus, the aim of this study was to assess OC risk in individuals with dental, prosthetic and functional CMI. Also, we examined CMI presence in relation to tumor size.

**Material and Methods:**

A case-control study was carried out from 2009 to 2013. Study group were squamous cell carcinoma cases; control group was patients seeking dental treatment in the same institution.

**Results:**

153 patients were studied (Study group n=53, Control group n=100). CMI reproducibility displayed a correlation coefficient of 1 (*p*<0.0001). Bivariate analysis showed statistically significant associations for all variables (age, gender, tobacco and alcohol consumption and CMI). Multivariate analysis exhibited statistical significance for age, alcohol, and CMI, but not for gender or tobacco. Relationship of CMI with tumor size showed no statistically significant differences.

**Conclusions:**

CMI could be regarded as a risk factor for oral cancer. In individuals with other OC risk factors, proper treatment of the mechanical injuring factors (dental, prosthetic and functional) could be an important measure to reduce the risk of oral cancer.

** Key words:**Oral cancer, risk factors, chronic mechanical irritation, tumor size, case-control study.

## Introduction

Carcinogenesis is multifactorial in humans. Tobacco and alcohol are often considered as the major risk factors for oral cancer (1). However, they could not cause the entirety of cancers, and also there are individuals unexposed to those factors that could develop malignant lesions. This fact implies that there are other factors in oral cancer, and among them, chronic mechanical irritation (CMI) has been mentioned.

CMI of the oral mucosa is the result of repeated injuring by the mechanical action of an intraoral injury agent. Defective teeth (malpositioned or with sharp or rough surfaces because of decay or fractures), ill-fitting dentures (sharp or rough surfaces, lack of retention, stability or overextended flanges) and/or parafunctional habits (e.g. oral mucosa biting or sucking, tongue interposition or thrusting), acting individually or together, could all be responsible for any mechanical irritation (2). CMI could generate changes in the healthy mucosa or intensify previous oral diseases (3). CMI produces several alterations related to its duration and intensity. Effects could range from a hyperproliferative epithelial response if the stimulus is mild (frictional keratosis), to several levels of tissue injury (atrophy, erosion, ulcer) if it is intense or is of longer duration (chronic traumatic ulcer), often with fibrous connective tissue growth (Reactive hyperplasia, e.g. Denture-induced fibrous hyperplasia) (4).

CMI has been considered as a potential risk factor for oral cancer (5), and even as a potentially malignant disorder (6), yet it is controversial because there is still scarce evidence to support this claim (7). Some authors have suggested that the relationship between OC and CMI could be the result of tumor’s growth; the larger the tumor, the more probability of being injured. Nonetheless, several cases of OC on the site of CMI because a broken tooth or a defective denture have been described (8,9). In addition, OC occurs mainly in locations that could be exposed to prosthetic or dental CMI, particularly in non-smokers without other risk factors (10). Early epidemiological studies dealing with that relationship had small samples or insufficient control of confounding factors, which hampered inferences (11). Velly *et al.* found a statistically significant risk of OC in association with history of oral sores secondary to ill-fitting dentures, but not with defective teeth (12). Lockhart *et al.* found examples of OC in relation to mechanical irritation from teeth or dentures, but obtained inconclusive results, suggesting that it might be a small sample. However, it should be noted that extra-oral malignancies were used as control, and among them, there are cancers that were later found to have relation with bad oral health (13). Rosenquist *et al.* found a significant association between OC risk and 5 or more defective teeth (14). Defective teeth have been pointed out by some authors as a common finding in OC patients (15). Also, it has been shown a significant statistical relationship between ill-fitting dentures and oral cancer (16). A recent meta-analysis showed the use of removable dentures itself does increase OC risk, and much more if they were ill-fitting dentures (17). Overall, the above-mentioned epidemiological studies support the notion that CMI of the oral mucosa could promote dysplasia and carcinogenesis, regardless of other risk factors. This effect could alter oral mucosa easily if it has been previously initiated by another carcinogen. This situation could be the case with oral submucous fibrosis, where CMI significantly increases epithelial dysplasia (18).

Most of the studies dealing with CMI and OC used prosthetic and dental variables, regardless that CMI effect could act independently of the causative factor. In other words, CMI could be considered as an OC risk factor, whether it is originated by dentures, teeth or functional factors. Only one cross-sectional study showed a statistically significant association between CMI and OC including all possible sources of mechanical irritation (dental, prosthetic and functional) (2). So, the aim of this case-control study was to assess OC risk in individuals with dental, prosthetic and functional CMI. Also, we examined CMI presence in relation to OC size.

## Material and Methods

- Design

A case-control study was carried out in Dentistry College of Universidad Nacional de Córdoba (Argentine) from 2009 to 2013. This study was approved by the Research and Ethics Committee of the Ministry of Health of the province of Cordoba (No. 1378) and informed consent forms were signed by all patients. All procedures performed in studies involving human participants were in accordance with the ethical standards of the institutional and/or national research committee and with the 1964 Helsinki declaration and its later amendments or comparable ethical standards. All examinations were carried out by previously calibrated professionals.

The Oral Cancer (OC) group were cases of squamous cell, in situ and verrucous carcinoma (ICD-10 C00-C06), confirmed histopathologically. Individuals with previous malignancies or oncologic therapy and lip vermillion cancer were excluded. Control group were patients with similar age range seeking dental treatment in the same institution and time period. The same exclusion criteria applied, and chief complaints due a CMI lesion were also excluded.

- Clinical Exam 

Epidemiological data such as age, gender, CMI, tobacco and alcohol consumption, were registered in a standardized form.

Tobacco consumption was recorded assessing how long the period of major consumption lasted, and the daily mean of smoking within that period; the number of cigarettes smoked before and after the period of major consumption, and how many years each part lasted. For each period, the daily mean was multiplied by the number of years that period lasted, and the result was then multiplied by 365 to obtain the subtotal of cigarettes smoked for each part. These subtotals were added up to obtain the total tobacco consumption, expressed in number of cigarettes. It was deemed as smoker when an individual smoked occasionally or regularly for more than a year.

Alcohol consumption was obtained ascertaining weekly volume consumed for each beverage type, expressed in liters. This value was multiplied by 52 to obtain annual consumption, which was subsequently multiplied by years of consumption for each beverage. Alcohol grams was obtained multiplying alcohol consumption by 8 and by alcohol concentration for each type of beverage (Beer 4.5%, Wine 13%, distilled liquor 30%). Subtotals for each beverage were finally added up to get total consumption in alcohol grams. It was deemed as drinker when an individual drank alcohol regularly for more than a year.

**Figure 1:**
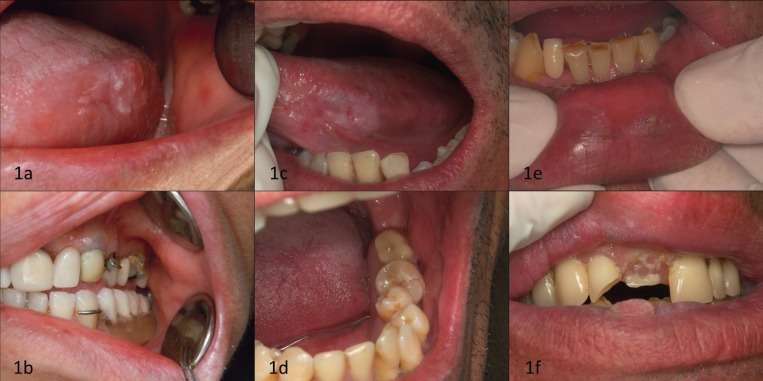
Clinical cases of oral cancer associated to chronic mechanical irritation. a,b) Female (53 years old), non-drinker and non-smoker, presenting a squamous cell carcinoma on the border of the tongue. Given the inverted overbite, the bicuspid proclination and lack of canine guide, the patient involuntarily bite the area for more than three years. c,d) Male (44 years old), non-smoker, drinker of more than 350,000 gr of alcohol. Lateral tongue border presents a verrucous carcinoma. This area was in relation to CMI originated by the lingual position of lower premolars and molars and a defective partial removable denture, both in contact with the lesion for more than four years. e,f) Male (55 years old), smoker of more than 200,000 cigarettes and drinker of more than 600,000 gr of alcohol. The patient had a leukoplakia without dysplasia. A year later, an upper removable denture was broken, and since then a verrucous carcinoma began its growth in the injured site.

CMI of oral mucosa was recorded as present (on clinically healthy mucosa or in association with pathologies) when any of the following conditions were found (2):

• Objective clinical lesion compatible with traumatic origin (e.g. erythema, atrophy, ulcer, keratosis, hyperplasia, indentation, fibrosis) with evolution of over a month; in relation to any dental, prosthetic or mechanical agent that should be in the oral cavity before the onset of the lesion. Also, the traumatic agent must be in direct contact with the lesion, during functional/parafunctional movements (such as swallowing disorders, tongue thrust, decubitus position, unilateral chewing, suction, denture stabilization using tongue, and so forth).

• Ulceration history caused by similar causes on the site of cancer onset with more than a month, in relation to similar factors (dental, prosthetic or functional).

• Oral cancer in relation to the aforementioned traumatic factors that should be present before the patient awareness of the lesion (tumor or ulcer). This data was gathered through oral inspection and anamnesis, taking special caution regarding recall bias. Figure 1 shows clinical examples of CMI’s associated carcinomas. Particularly 1C and 1D offers a good illustration of this point, where a verrucous carcinoma of left border and ventral surface of the tongue can be seen in contact with both teeth and removable dentures. Teeth are in a manifest malposition (particularly lower left 2nd molar and 2nd bicuspid). Although the patient could not remember with certainty when dental IMC started (originated because of the malposition), it is indeed possible to estimate since when the injuring condition exists. The 2nd molar began to move mesially when the 1st molar was removed, and the patient clearly remembers that happing at his 20 years. At present the patient is 44 years old, so it could be expected that the injuring condition existed for at least 24 years, a time period obviously longer that the development of the verrucous carcinoma. In all the IMC cases a similar reasoning was made in order to assess the time of the injuring factor.

CMI factors were registered in three groups: dental (tooth malposition, diastema, sharp or broken tooth, sharp or rough fillings or fixed prosthesis), prosthetic (sharp or rough dentures, defective retainers, overextended flanges, denture without stability and/or retention) and functional (tongue interposition, lip/cheek/tongue biting or suction, dentures stabilization with tongue, etc.). CMI factor time spam was registered in months. Additionally, CMI was recorded double-blind on 20 patients by two operators to test reproducibility.

Additionally, in the OC group tumor size was registered according to TNM classification, to compare tumor size with CMI presence (19).

- Statistical Analysis 

Data were described by their absolute and relative (%) frequencies. Quantitative variables were analyzed through Mann-Whitney. Associations were initially evaluated by bivariate analysis. Logistic Regression model was built combining age, gender, CMI and smoking/alcohol consumption habits. The diagnostic accuracy of the model was assessed by the Area Under of the Receiver Operating Characteristic (AUC of ROC curve) estimated by non-parametric methods. Statistical significance was set at *p*<0.05.

The Kappa coefficient was calculated to evaluate the concordance between professionals, setting a value ≥0.6 for concordance. All analyses were carried out with Infostat software (v. 2015, www.infostat.com.ar).

## Results

OC group (n= 53) had 29 males and 24 females (1.2 ratio), whereas Control group (n=100) had 36 males and 64 females. Study group mean age was 63 years, and in Control group was 46.7 years. Demographic and clinical data of the case and control groups are shown in  Table 1.

**Table 1:**
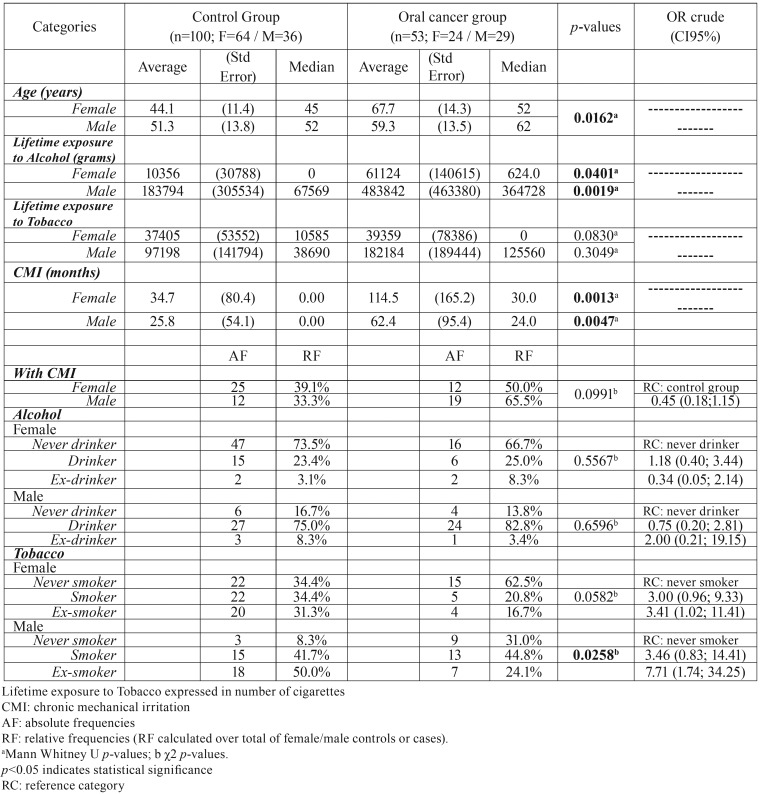
Demographics and clinical variables.

Predominant cancer was squamous cell carcinoma (85%, n=45), the rest were Verrucous carcinoma (11%, n=6) and in situ carcinoma (4%, n=2). Regarding location, tongue (51%, n=27) was the most affected, followed by gingiva and alveolar ridge (15%, n=8), buccal mucosa (13%, n=7), floor of the mouth (9.5%; n=5), hard and soft palate (9.5%, n=5), and labial mucosa (2%, n=1). CMI associated lesions on control group were as follows: red lesions (erythema/atrophy, n=20); tongue indentations (n=9); frictional keratosis (n=5), petechiae (n=5), fibrous hyperplasias (n=5), morsicatio buccarum (n=2), chronic traumatic ulcer (n=1) and one case of an plaque-like oral lichen planus aggravated by CMI that improved when CMI was removed. Of the 36 cases of the control group, 11 showed more than one CMI associated lesion. CMI reproducibility displayed a correlation coefficient of 1 (*p*<0.0001).

Alcohol consumption, age and CMI (in this case recorded in months of exposition) for both genders showed a statistically significant difference between OC and Control group. However, tobacco consumption showed no differences between groups (Male: *p*=0.3049; Female *p*=0.083).

Oral cancer occurrence was higher in association with CMI regardless tobacco and alcohol consumption ( Table 2).

**Table 2:**
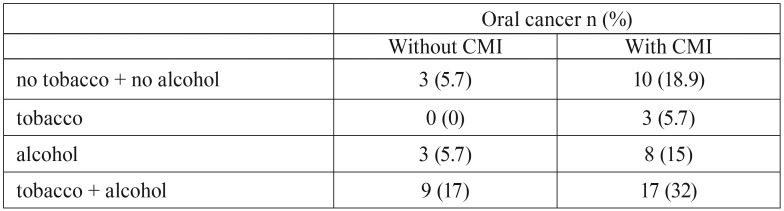
Frequency of CMI in oral cancer group according to tobacco and alcohol consumption.

Bivariate analysis showed statistically significant associations for all five variables: age, gender, tobacco and alcohol consumption and CMI ( Table 3). However, it should be noted that 45% (n=24) of the study group never smoked. CMI presented a significant statistical association with OC stratifying in never drinker (ND), drinker (D), smoker (S) and never smoker (NS).

**Table 3:**
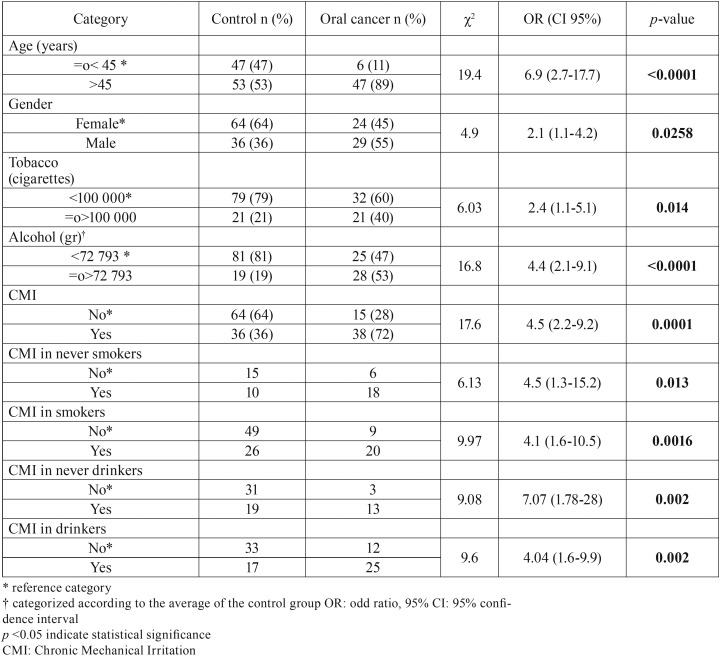
Bivariate analysis.

**Figure 2:**
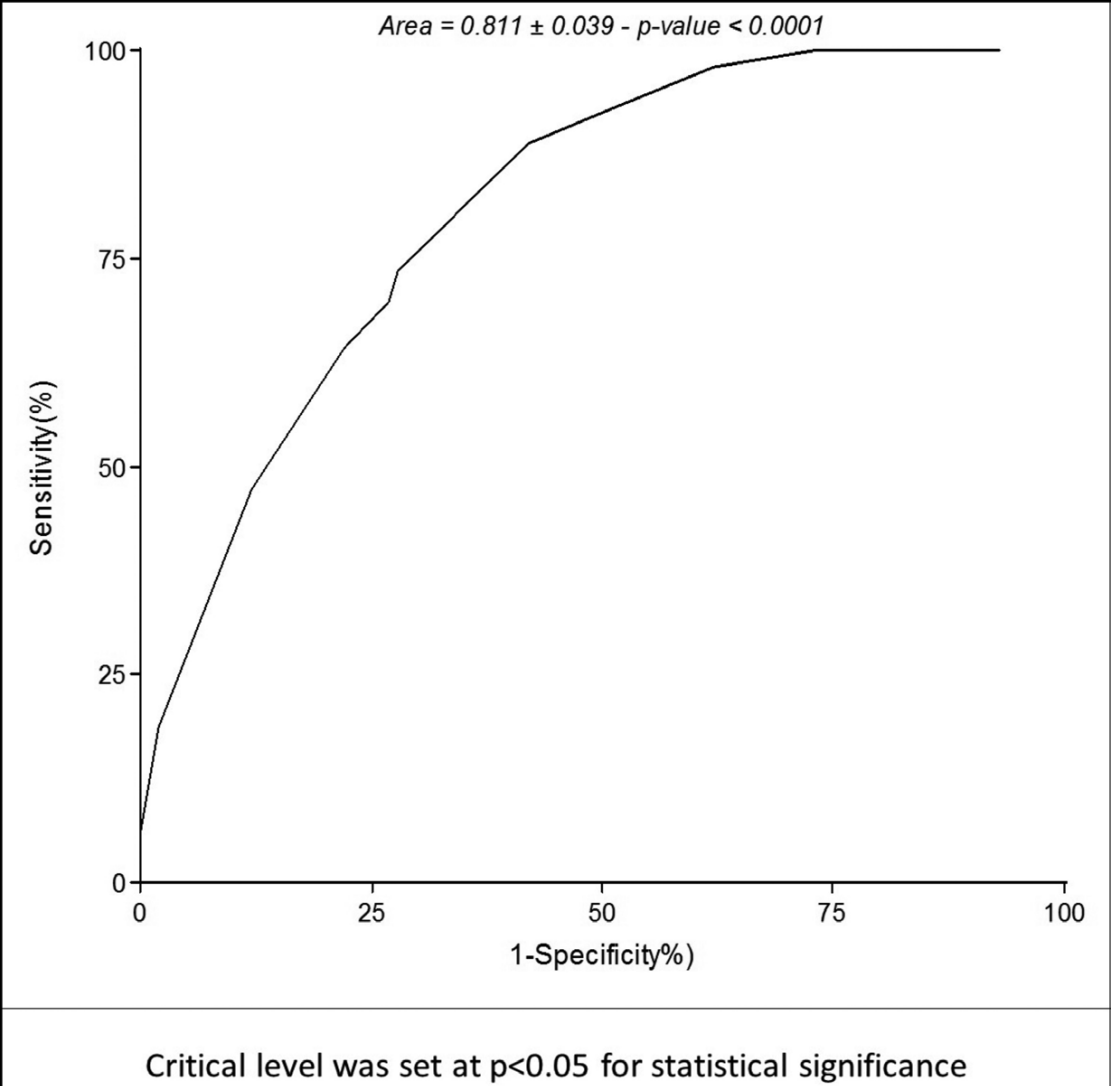
Area under ROC curve built by predictive values estimates of logistic regression

**Figure 3:**
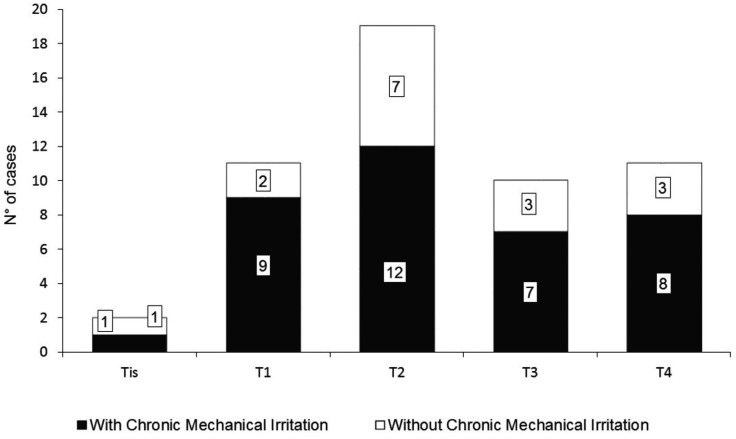
Tumor size distribution according to TNM classification and CMI.

On the other hand, multivariate analysis exhibited statistical significance for age, alcohol, and CMI, but not for gender or tobacco ( Table 4). Logistic regression showed an association with OC for age and CMI for NS, S, D and ND. OR for CMI was 4.95 (1.21-20.22; *p*=0.026) for NS, 3.87 (1.32-11.38; *p*=0.014) for S, 10.6 (1.64-68.83; *p*=0.026) for ND, and 3.28 (1.22-8.78; *p*=0.014) for D. AUC of ROC curve is shown in figure 2,3.

**Table 4:**
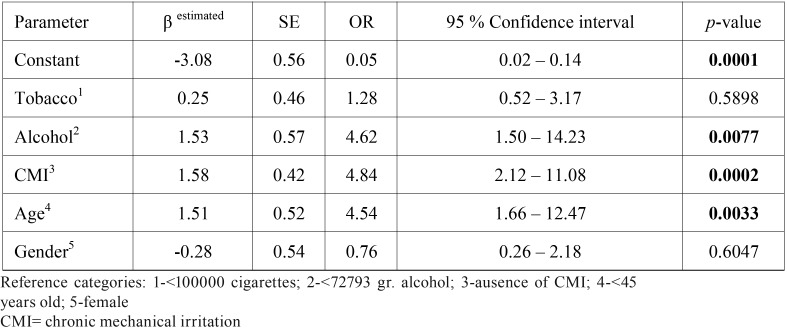
Logistic Regression.

CMI was examined using X2 in relation to number of defective teeth, excluding completely edentulous individuals, showing a 1.51 OR (CI 95%, 0.72-3.2, *p*=0.269).

Tumor size and its association with CMI is depicted in figure 3. Considering Tis, T1, and T2 as small, they added up to 60% of the study group, whereas large size malignancies (T3 and T4) account for the rest. Relationship of CMI with tumor size was studied comparing small tumors (Tis, T1 and T2) versus large tumors (T3 and T4), revealing an OR of 0.88 (CI 95%, 0.26-2.94), *p*=0.84. Tis-T1 versus T2 versus T3-T4 were also compared (*p*=0.69), meaning that there were no differences in CMI occurrence in relation to tumor size.

## Discussion

Previous studies dealing with the relationship between oral cancer and CMI employed dental or prosthetic variables, but without assessing them together. Thus, it is essential to define a clear and reproducible criterion for CMI that includes all the potential CMI factors. We applied one based on the protocol proposed by Piemonte *et al.* (2). Identifying CMI offered a high a correlation coefficient, which suggests that the proposed method is repeatable.

CMI occurrence on Control group was 36%, which emphasizes CMI as a common oral condition. Nonetheless, in the Study group, its appearance almost doubled (72%), representing one of the most prevailing risk factors, even more than tobacco and alcohol. Regardless of smoker and/or drinker conditions, CMI was found in significant statistical relation with OC, in both the bivariate and the multivariate analysis. These details could suggest that CMI may play a role not only as an oral cancer risk factor but also could be considered as a potentially malignant disorder of the oral mucosa (PMD).

Nevertheless, inclusion as a PMD could not be done employing the same requisites for other disorders –e.g. leukoplakia-. This is due to methodological restrictions posed by the developmental and therapeutic features of CMI lesions. Mechanical irritation usually produces painful ulcers that are quickly submitted to consultation and treatment. For that reason, persistence ensues only in a minor number of cases, possibly because of decrease or loss of sensitivity. Furthermore, unlike another oral PMD like lichen or leukoplakia, it is not possible to carry out study cohorts of CMI. Ethically, it would not be possible to leave a patient with CMI untreated to study its evolution. When the causative factor of a CMI lesion is addressed it typically heals, disrupting microenvironment inflammation conditions that foster carcinogenesis (20). If cancer develops from a CMI lesion, likely the original traumatic lesion would be modified –if not completely disappeared- because of tumor growth. This situation is not uncommon due to delay that arises in oral cancer diagnosis (21). Therefore, only initials cancers originated on CMI lesions would be included, which would be an inclusion bias, lowering CMI associated cancers recording.

The absence of evidence on CMI and oral cancer might mean that a causal relationship could not be proven yet mainly to the methodological issues mentioned. To overcome this limitation, some studies assessed oral cancer risk and recurrent sores by ill-fitting dentures, showing significant association (16). In the present work, we determined the presence of a CMI condition before the onset of the tumor to assess CMI. Studies dealing with the relationship between CMI and oral cancer assess prosthetic and dental variables separately (22). The mechanical damaging effect is produced independently of the material of the CMI factor (23). Whether it is acrylic resin, metal alloy, enamel or dentine, it only needs to be harder than oral mucosa to induce injury. Piemonte *et al.* indicated CMI as an oral cancer independent risk factor, taking into account dental, prosthetic and also functional factors (2). This last one promotes mucosal damage increasing contact and strength of a mechanical cause, e.g. sharp teeth. Hence, it may be needed more than a defective tooth/denture to produce a mechanical irritative lesion, but also a functional alteration (swallowing disorders, tongue biting, etc.) that increases contact (3). This could explain why defective teeth, a rather common feature in populations with limited access to dental services, has not been associated with oral cancer in epidemiological studies. In the present study, defective teeth itself was not in relation to CMI. This fact further supports the notion that more than defective teeth or denture is needed to generate CMI, emphasizing the importance of functional factors (3). Therefore, it is important to note that defective teeth/dentures itself is an insufficient criteria to assess mechanical irritation because the sole presence of CMI factors does not always produce a lesion.

The analysis of CMI and tumor size is also noteworthy. It has been suggested that mechanical irritation could arise because of tumor growth, describing CMI as a consequence of tumor size. Even if this could happen on same occasions, the CMI criteria used in this study helps to avoid this bias, because it is stated that the CMI agent must present before the onset the lesion. Moreover, the absence of statistically significant differences in CMI occurrence according to tumor size suggests that not necessarily cancer growth induces more CMI (Fig. 2).

Not much is known about the potential pathogenic events linking CMI with oral carcinogenesis. In animal experiments of chemical carcinogenesis, repeated mechanical irritation of the oral mucosa pinpoints tumor site, prompts an increase in incidence and malignancy index, and reduces latency time (24). It is suggested that breaks in mucosal integrity as a result of CMI could foster carcinogen absorption. Similarly, the increase in cell proliferation prompted in wound healing could place cells in a higher risk of random or carcinogen-induced mutations, promoting malignant alterations (25). Particularly when CMI produces an open wound (namely a chronic traumatic ulcer), it could meet the requirements to act as a promoter cofactor in malignant transformation (24).

The available evidence suggests that CMI could play a role at least as a tumor promoter, but the pathological mechanisms are still not completely understood. If CMI could only act increasing exposure or penetration of carcinogens, its role would be tied to the absorbed carcinogen. Thus, if CMI only allowed more tobacco substances to be absorbed, it should have a statistically significant association with oral cancer in smokers, whereas its effect in non-smokers should be non-existent, and the same could be said regarding alcohol consumption. However, in our study, CMI consistently showed a statistically significant association with oral cancer in all groups disregarding alcohol or tobacco consumption. Consequently, the promoter role may be exerted through other ways, and among them, it is worth mentioning oxidative and epigenetics effects due to chronic inflammation.

Nowadays it is widely accepted that recurrent or persistent inflammation could predispose to cancer. Recurrent of persistent inflammation due to infections, chemical substances, radiation, mechanical irritation or autoimmune diseases may induce or promote carcinogenesis by DNA damage, inciting cell proliferation, and the release of cytokines and growth factors (26). Most of the inflammation-associated tumors are of epithelial origin. Some well-known examples include chronic inflammatory bowel diseases and colon carcinoma, chronic inflammatory injury due to gastroesophageal reflux and esophageal cancers among others (27).

The existence of chronic inflammatory disorders that do not have an infective cause and are associated with the development of tumors, strongly suggests that the inflammatory process itself provides the environment for the development of malignancy (28). Chronic inflammation produces repeated cycles of cell injury and compensatory proliferation. The increase in a cell undergoing division turn them susceptible to DNA damage, also promoting malignant cell growth (29). The overexpression of several mediators in chronic inflammation offers an explanation of the potential role of chronic inflammation in cancer initiation, promotion, conversion and progression (28).

On animal models, it has been showed that mechanical irritation aids HPV penetration and stimulates reactivation (30–32). Such potential interaction between CMI and HPV on oral carcinogenesis has not been studied yet, even when both are common conditions in oral cancer patients.

In our study, multivariate analysis showed that tobacco consumption does not have a statistically significant association with OC. This is a result of particulars features of the studied sample, where almost half of it has never smoked. Thus, this somehow contradictory outcome stresses CMI relevance, not only as tobacco co-factor but also as an independent risk factor for never smokers. It is noteworthy that the association between OC and tobacco consumption has been historically studied without been adjusted according to CMI, an even another variables such as periodontal disease and teeth loss. On papers with meta-analysis revising association of OC with CMI, periodontal disease and teeth loss, the selected original studies were adjusted to alcohol and tobacco, but not inversely (22,33,34). So, the present work would be the first offering a multivariate analysis of oral cancer including alcohol and tobacco consumption adjusted with CMI, therefore taking into account the multifactorial etiology of OC. Even with the limitations imposed by our study, it would be needed to study in deep OC and tobacco adjusted with variables indicative of oral status, not just CMI.

Results from case-control studies, in general, should be interpreted with caution because the pattern of recall bias frequently encountered in such design tends to inflate the estimated risk attributed to the exposure under investigation. Some methodological strategies can minimize recall bias, and some of these strategies were used in this work: choosing newly diagnosed cases, using standardized data collection protocols, applying the instrument at similar timing in both study groups, giving the participants enough time before answering, and verification of exposure reported-data by using a reference criterion or another source of reported-data.

It should be duly noted that in our study the Control group was composed of individuals seeking dental treatment, many of them having lost teeth or using removable dentures, which offers proper conditions for CMI occurrence. Therefore, a selection bias could have happened, because people that had less need of dental treatment –and consequently, the less potential risk of CMI- were not included. Wich could suggest that CMI is undervalued as a risk factor, and its relevance in oral carcinogenesis might be of even more importance.

## Conclusions

Our study reinforces the hypothesis that CMI could act at least as a co-factor in oral carcinogenesis. In individuals with other OC risk factors, proper treatment of the mechanical injuring factors of oral mucosa (dental, prosthetic and functional) could be an important measure to reduce the risk of oral cancer.

Even so, evidence of the association between OC and CMI is still scarce. Thus, more epidemiological and biomolecular studies that delve into CMI and oral carcinogenesis are needed.
